# Eradication of Yaws: Historical Efforts and Achieving WHO's 2020 Target

**DOI:** 10.1371/journal.pntd.0003016

**Published:** 2014-09-25

**Authors:** Kingsley Asiedu, Christopher Fitzpatrick, Jean Jannin

**Affiliations:** Department of Control of Neglected Tropical Diseases, World Health Organization, Geneva, Switzerland; University of Tennessee, United States of America

## Abstract

**Background:**

Yaws, one of the 17 neglected tropical diseases (NTDs), is targeted for eradication by 2020 in resolution WHA66.12 of the World Health Assembly (2013) and the WHO roadmap on NTDs (2012). The disease frequently affects children who live in poor socioeconomic conditions. Between 1952 and 1964, WHO and the United Nations Children's Fund (UNICEF) led a global eradication campaign using injectable benzathine penicillin. Recent developments using a single dose of oral azithromycin have renewed optimism that eradication can be achieved through a comprehensive large-scale treatment strategy. We review historical efforts to eradicate yaws and argue that this goal is now technically feasible using new tools and with the favorable environment for control of NTDs. We also summarize the work of WHO's Department of Control of Neglected Tropical Diseases in leading the renewed eradication initiative and call on the international community to support efforts to achieve the 2020 eradication goal. The critical factor remains access to azithromycin. Excluding medicines, the financial cost of yaws eradication could be as little as US$ 100 million.

**Conclusions:**

The development of new tools has renewed interest in eradication of yaws; with modest support, the WHO eradication target of 2020 can be achieved.

## Introduction

The endemic treponematoses, which comprise yaws, endemic syphilis (bejel) and pinta, are a group of chronic bacterial infections caused by members of the genus *Treponema* [1]. *Treponema pallidum pertenue*, *Treponema pallidum endemicum*, and *Treponema pallidum carateum* are the causative agents of yaws, bejel (or endemic syphilis), and pinta, respectively. Although not fatal, these infections cause painful and sometimes disfiguring lesions of the skin, cartilage, face, soft tissue of the mouth, and bones. In about 10% of chronic untreated cases, permanent disability and associated stigma may result. The endemic treponematoses and sexually transmitted syphilis cannot be distinguished by serological tests and all respond to treatment with injectable benzathine penicillin [2]. There is no vaccine to prevent the diseases. In 1950, the World Health Organization (WHO) estimated that 160 million people were infected with yaws, 1 million with endemic syphilis, and 0.7 million with pinta [3]. The toll of these diseases at that time was huge; more than 40 million people (mostly yaws cases) suffered gross destruction of tissue, joints, and bones, and facial disfigurement.

Yaws, the most prevalent of these three diseases, is found primarily in poor rural communities in warm, humid, and tropical forest areas of Africa, Asia, Latin America, and the Pacific. Children aged less than 15 years who live in poor socioeconomic conditions constitute the main reservoir of infection; transmission occurs through direct skin contact with the fluid from an infected lesion. Although yaws-like lesions have been found in primates in jungles in Africa, it is unclear if they can transmit the disease to humans [4]. The incubation period for yaws is estimated to be 9–90 days (with an average of 21 days).

Bejel, conversely, is found in dry and arid environments in Africa and the Middle East. Children aged under 15 years are the most affected; transmission occurs through the sharing of contaminated drinking cups. Bejel appears to be rare with a few cases occasionally reported [5].

Pinta is found only in Latin or South America. It affects both children and adults, but those aged 15–30 years are more affected. Transmission occurs through direct skin-to-skin contact. The incubation period is 2–3 weeks. Unlike yaws, no disability or complication occurs; hypopigmented skin is the only main residual effect of the disease, pinta is regarded as the mildest form of the treponematoses. There is no recent information on the disease.

The distinctive clinical features of endemic treponematoses have recently been described [2]. The article focuses on the evolution of yaws eradication, the most prevalent of these diseases, although its content is applicable to both bejel and pinta.

The aim of this article is to provide a better understanding of the historical efforts to achieve yaws eradication. Lessons learned from the past are reviewed and acknowledged and key matters that are required for the renewed eradication efforts are also discussed.

We reviewed published articles in PubMed, WHO library databases, and unpublished reports from 1 January 1950 to 30 October 2013, using the terms “yaws,” “pian,” “bejel,” “pinta,” “endemic treponematoses,” “Treponema pallidum,” and “neglected tropical diseases.” We searched articles that had data on historical conference proceedings, eradication programmes, and treatment policies. We consulted experts and requested additional data from investigators to evaluate the progress of the new eradication strategy (2012) and to establish preliminary investment benchmarks for yaws eradication.

## Historical Eradication Efforts

**Figure 1:**
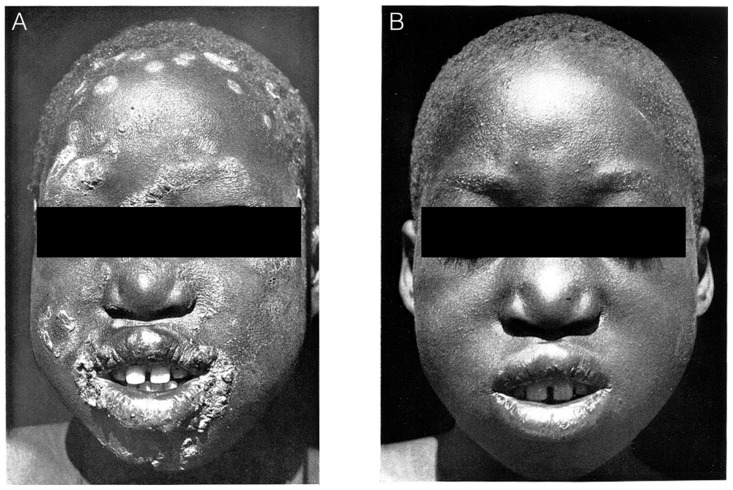
Results of treatment with a single injection of benzathine penicillin in the 1950s. Panel A shows a patient with yaws lesions (papilloma) on the face before treatment. Panel B shows the same patient two weeks after treatment with a single injection of benzathine penicillin.

In 1948, when WHO was established, endemic treponematoses were among the major public health problems that the new health agency had to deal with. The large geographical distribution and high burden of yaws before the 1952 mass treatment campaigns justified the urgency and actions taken. For example, in 1936 in the then Gold Coast (now Ghana), yaws constituted 62.7% of all infectious diseases treated in government health facilities compared with 20.3% for malaria [6]. Similarly, in Nigeria in 1935, among infectious diseases treated at government health facilities, yaws constituted 47.76% compared with 15.61% for malaria [7]. The World Health Assembly resolution WHA2.36 [8] in 1949 to support control of endemic treponematoses was therefore timely and appropriate. The initial WHO-assisted pilot projects [9] to introduce penicillin in mass treatment campaigns in Bosnia, Haiti, Indonesia, the Philippines, and Thailand were rapid and remarkably successful. The spectacular and visible results achieved with single-dose treatment (Figure 1) helped to reinforce community cooperation in the campaigns.

In March 1952, WHO organized the first international conference [10] on yaws in Bangkok, Thailand, attended by 70 participants from 23 countries. The objectives of this meeting were to assess the global status of yaws and to share the experiences gained in pilot countries with other endemic countries. In November 1955, WHO convened a second international conference on yaws in Enugu, Nigeria, attended by 53 participants from 30 countries [11]. Africa was chosen as the venue because it was the home to about half of the estimated 50 million yaws cases in the world at that time. The venue in the eastern part of Nigeria was also chosen because of active and successful yaws control activities [12]. The objectives of the conference were to review the progress made and provide guidance to health authorities of the endemic countries. Basic operational principles to guide yaws eradication were established, noting that success would depend on 100% treatment coverage of both active clinical disease and latent infections; anything below 90% was considered inadequate. In 1956, the Pan American Sanitary Bureau, now Pan American Health Organization (PAHO), organized a seminar on the eradication of endemic treponematoses in the Americas at Port-au-Prince, Haiti [13]. At this meeting, which was attended by 48 participants, the practicability of yaws eradication was stressed, and a plan for a coordinated implementation in the region was agreed upon.

During 1952–1964, WHO and the United Nations Children's Fund (UNICEF) supported mass treatment campaigns using injectable penicillin in 46 countries. About 300 million people were screened, and over 50 million cases and contacts were treated. By the end of the campaign, the global burden of cases of endemic treponematoses was estimated to have reduced by 95%, to just 2.5 million cases. The implementation of this highly vertical programme also contributed to delivering much needed healthcare to affected communities [14]. Where possible, other diseases common in the communities—such as malaria, sleeping sickness, leprosy, smallpox, and yellow fever—were addressed by the yaws team, highlighting the historic concept of integrated public health interventions at the community level. Based on the experiences gathered in the field, Hackett CJ and Guthe T summarized the principles of yaws eradication to guide all those involved in the planning and implementation of yaws eradication campaigns [15].

The success of these campaigns in significantly reducing the global prevalence of yaws and other endemic treponematoses was credited as one of the greatest public health achievements in the history of WHO [16]. For UNICEF, yaws eradication was characterized as one of the most profitable investments it made, considering the per capita cost and amount of suffering alleviated [9]. Unfortunately, the vertical programmes were gradually dismantled in favour of their integration into the weak primary health care systems, confident that these would suffice to identify and treat the remaining 5% of cases. Ultimately, the lack of continued surveillance and waning of commitment and resources led to the resurgence of yaws in West Africa, Asia, and the Pacific in the late 1970s. The World Health Assembly consequently adopted resolution WHA31.58 in 1978 [17]. This resolution requested Member States 1) to formulate and implement integrated treponematoses control programmes with particular emphasis on active surveillance so as to interrupt transmission of the diseases at the earliest possible time in the areas where they are still endemic and to prevent their recurrence in areas from which they have been eliminated or where they have never been endemic, and 2) to report regularly to WHO on the current epidemiological situation of endemic treponematoses.

In response to this resolution, control activities were renewed in a number of countries, notably West Africa in the early 1980s. In Ghana, a combined yaws and yellow fever project was implemented in 1981 with funding from the United States Agency for International Development (USAID), WHO, UNICEF, and the European Economic Community (EEC) [18]. Efforts were made to galvanize support from the international community and regional bodies for the eradication effort. In 1980, the Fogarty International Center, United States convened an expert meeting to review different diseases and their suitability for eradication [19]. Three diseases—measles, poliomyelitis, and yaws—were considered suitable for eradication or at least elimination at the regional level, among which yaws was considered to be the best candidate for eradication. As a follow-up to this 1980 meeting, the Fogarty International Center, together with ten other organizations, sponsored an international symposium on yaws and other endemic treponematoses in 1984 to review the status of these diseases, and to consider strategies, technologies, and research needed for their control and eventual eradication. This meeting, which was held at the Pan American Health Organization office in Washington, D.C., U.S. was attended by over 60 participants [3]. Regional meetings then followed in Cipanas, Indonesia (1985) [20], Brazzaville, the Congo (1986) [21], and Amman, Jordan (1986) [22] to draw up plans for interrupting transmission. The regional meeting on yaws and pinta for the Americas was replaced by a consultant's evaluation of the situation in 1987 (PAHO Internal document) [23]. However, the organization of international and multiple regional meetings was not sufficient enough to revive the global interest in control and eradication of these diseases.

## Historical and Current Geographical Distribution of Yaws

**Figure 2:**
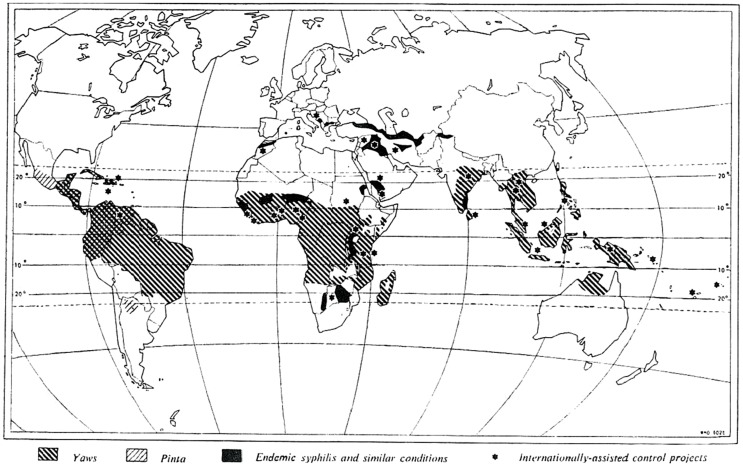
Global distribution of endemic treponematoses in the 1950s. The figure shows that endemic treponematoses (yaws, bejel, and pinta) were widespread between latitudes 20 North and South; yaws was the most prevalent of the three diseases.

**Figure 3:**
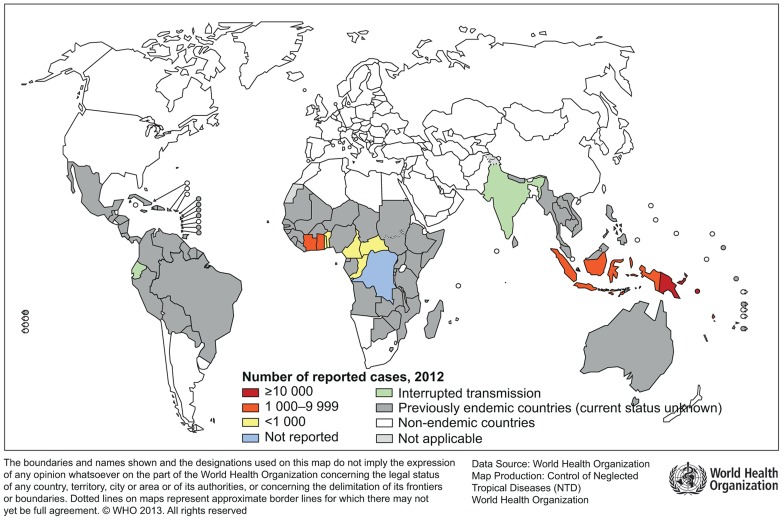
Global distribution of countries actively reporting yaws cases in 2012. The figure shows the 12 currently endemic countries, two countries that have interrupted transmission and 71 countries for which the current status is unknown. Source: Global Health Observatory: http://apps.who.int/gho/data/node.main.NTDYAWSEND?lang=en.

A review of the historical and current literature [24] from 1950 to 2013 indicates that at least 85 countries [25] have ever reported yaws (Table 1, Figure 2, and Figure 3). However, WHO's technical assistance in the 1950s to 1960s was provided to only 46 of these countries. Since 1990, formal reporting of yaws from a number of countries to WHO stopped. The Organization also did not have any formal system to verify interruption of transmission and certify countries. Only 14 countries kept yaws activities on their public health agendas, of which 2 countries (Ecuador [26] and India [27], [28]) reported interrupting transmission of the disease in 2003; formal verification by WHO is needed. The remaining 12 countries need technical assistance and resources to eradicate the disease.

**Table 1:** Status of endemicity (known and unknown) in four out of six WHO regions in 2012 [25].

WHO region	Endemic (Status known)	Previously endemic (Status unknown)	Total
African	8	30	38
Americas	1	24	25
South-East Asia	2	5	7
Western Pacific	3	12	15
**Total**	**14**	**71**	**85**

12 endemic countries: Benin, Cameroon, Central African Republic, Congo, Côte d'Ivoire, Democratic Republic of the Congo, Ghana, Togo, Indonesia, Papua New Guinea, Solomon Islands and Vanuatu

Two countries interrupted transmission: Ecuador and India

Source: Global Health Observatory: http://apps.who.int/gho/data/node.main.NTDYAWSEND?lang=en

For the ≥71 countries where no recent data are available, efforts are needed to verify the presence or absence of the disease. However, in view of the social and economic development of some of these countries since 1950, and comparatively more people living in urban areas, it is possible that the geographical distribution of the disease has considerably shrunk within and across countries compared with the situation in the 1950s. In particular, information on yaws and pinta in the region of the Americas is very limited; the last report is dated 1993 [29]. Opportunities for surveys (clinical and serological) to determine any ongoing transmission, where feasible, may be incorporated into other large-scale disease control programmes that target children aged under 15 years to reduce operational costs.

## Historical Treatment Policies

The historical mass treatment policies that formed the basis of yaws eradication were developed during the second international conference on yaws in Enugu, Nigeria in 1955. Experience had shown that it is only when active clinical cases and incubating and latent infections are simultaneously treated that interruption of transmission can be achieved. Treatment policies were based on the prevalence of clinically active yaws in the entire population of a village to determine the policy to apply whereby (i) in areas where prevalence exceeds 10%, the entire population should be treated with benzathine penicillin, (ii) in places where prevalence is 5%–10%, all children aged under 15 years and close contacts should be treated, and (iii) in areas where prevalence is less than 5%, only household and other close contacts should be treated. Although contacts were defined as people having regular person-to-person interaction with patients with active infectious clinical yaws, it was difficult to fully define the extent of contacts and to treat all. Hence, the application of the second and third policies was unlikely to deal with all potential incubating and latent infections; and without very frequent resurveys (difficult and costly), it was impossible to interrupt transmission. The general consensus at the Enugu conference was to use total mass treatment even in areas where prevalence is lower than 10%.

**Figure 4:**
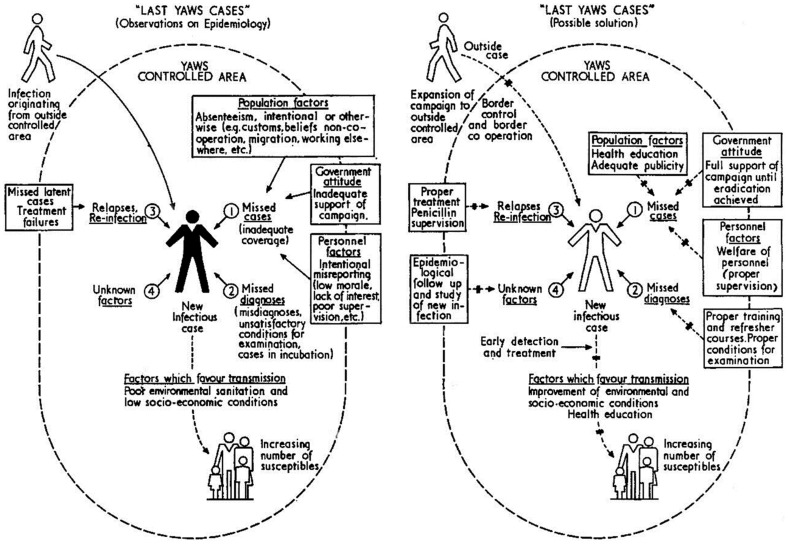
Yaws control: surveillance phase in the 1950s. The figure shows the steps taken in the 1950s to address factors and problems that could undermine the eradication effort during the post mass treatment surveillance phase [9, page 17].

Criteria for discontinuing mass treatment and routine population resurveys were established in 1960 to be when (i) at least 80% of the population has been seen in the last re-survey, (ii) the prevalence of active yaws is ≤2%, and (iii) the prevalence of infectious yaws is ≤0.5%. A surveillance system was then established through the local health facilities (rural health centers or health posts) supplemented by periodic school surveys with focus on children who are at the highest risk of infection. Key problems that could be encountered during the post mass treatment surveillance phase were identified and possible solutions were also proposed (Figure 4) [9].

## Technical Feasibility of Yaws Eradication

**Figure 5:**
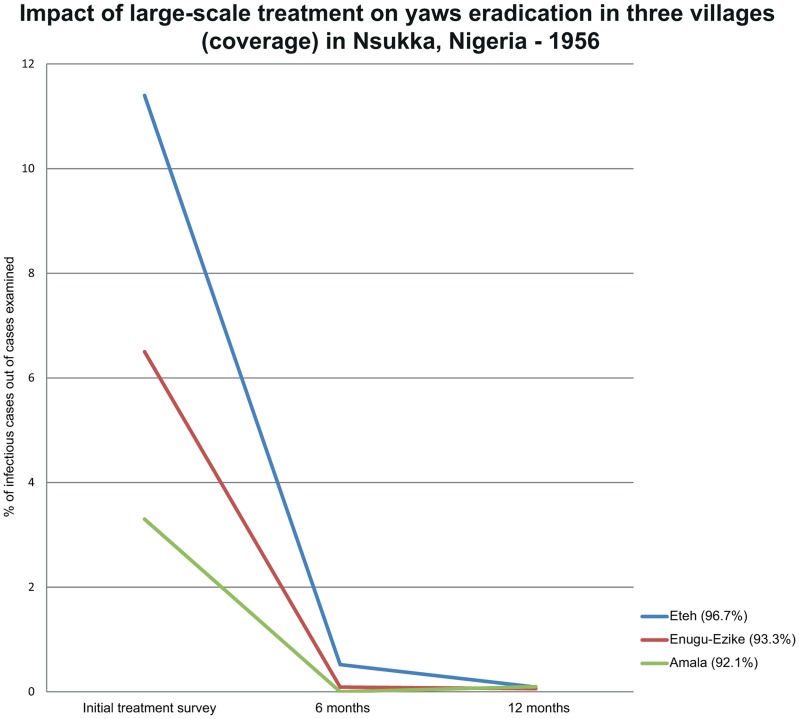
Impact of large-scale treatment on yaws eradication in three villages (coverage) in Nsukka, Nigeria in 1956. The figure shows the rapid decline in the prevalence of yaws within 12 months following large-scale treatment of endemic communities and the high coverage achieved.

Following successful experience in pilot projects, the second international conference on yaws in Enugu, Nigeria in 1955 embraced an ambitious plan for scaling up yaws control to elimination, particularly in Africa, and the ultimate goal of global eradication. Experience in the 1940s and 1950s of eradicating yaws in Haiti [30] and Nigeria [12] (Figure 5) and endemic syphilis in Bosnia and Yugoslavia [31] had shown that a single injection of long-acting penicillin coupled with treatment coverage exceeding 90% rapidly reduces the burden of the disease within 12 months. In Indonesia, where selective treatment policy of patients combined with regular resurveys was used, the rate of reduction in prevalence was not impressive [32], and it took 2–3 years to reach the same post-treatment prevalence that was achieved within one year in Nigeria.

The experiences gathered were used to make informed, evidence-based choices to move from yaws control to progressive eradication. The WHO Expert Committee on Venereal Infections and Treponematoses in 1960 [33] set two criteria for the eradication of yaws from a public health perspective.

1. Epidemiological eradication: was considered as the intermediate stage to complete eradication, defined as the absence of an indigenous infectious case in the population for three consecutive years. The basis of findings include information gathered from four sources: (i) all medical centres in the country where proper records of cases of the disease are kept, (ii) biannual medical examinations of all schoolchildren, (iii) annual surveys of randomly selected villages remote from medical facilities, schools, and towns, and (iv) reported from any reliable source of information.
2. Complete eradication: was considered as the final stage of achievement of eradication (interruption of transmission), defined as the absence of an indigenous case in the population for three consecutive years, with information from all the above sources having been considered and no seroreactor in the age group under five years having been found.

After the successful eradication of smallpox in the 1970s, attention was again refocused on yaws eradication [34]. Many experts believed it to be an attainable goal that should be pursued. At the International Symposium on Yaws and Other Endemic Treponematoses in 1984, the feasibility of eradication of yaws was again considered to be technically feasible and achievable: “*If the eradication of yaws can be accomplished, it should be done to reduce the suffering that is associated with the disease*”, William Foege [35].

In 2011, the WHO Strategic and Technical Advisory Group for Neglected Tropical Diseases reviewed the 17 NTDs and their suitability for elimination or eradication. In considering the current knowledge and the available tools, it recommended that yaws be targeted for eradication, and this was included in the WHO NTD roadmap of 2012 [36].

In 2012, the 20^th^ meeting of the International Task Force for Disease Eradication (ITFDE) [37] examined the recent developments in yaws, including new tools (single-dose oral azithromycin, rapid dual platform point-of-care (POC) syphilis test, and polymerase chain reaction (PCR) technology to monitor resistance) and lent its support for the renewed yaws eradication effort. However, ITFDE made some recommendations to WHO (Box 1). Most of these recommendations are being addressed.

Finally, in 2013, the World Health Assembly adopted resolution WHA66.12 [38] on all 17 NTDs, which targets the eradication of dracunculiasis (2015) and yaws (2020).

## Renewed Eradication Effort and WHO's Response

In 2006, WHO created the Department of Control of Neglected Tropical Diseases. The initial list of diseases did not include yaws, bejel, and pinta [39]. However, following the reports of increasing cases from its African, South-East Asian, and Western Pacific regions, WHO organized a three-day meeting in 2007 [40] to review the current situation and devise ways forward. The meeting recommended, as a first step, that the endemic treponematoses be included in the list of NTDs. This was done, and since then WHO has sought to highlight the problem of yaws on the international public health agenda. The interruption of transmission of yaws in India in 2003 and subsequent declaration of the elimination of the disease in 2006 [27], [28] provided an impetus to the renewed eradication initiative (Box 2). India is the second most populous country (>1 billion) in the world and one of the fastest growing economies. Together with smallpox and dracunculiasis, yaws and, recently, polio now belong to the public health history of the country. These achievements, despite its huge population, serve as a motivation for other countries to demonstrate that some carefully selected infectious diseases can be eradicated.

**Figure 6:**
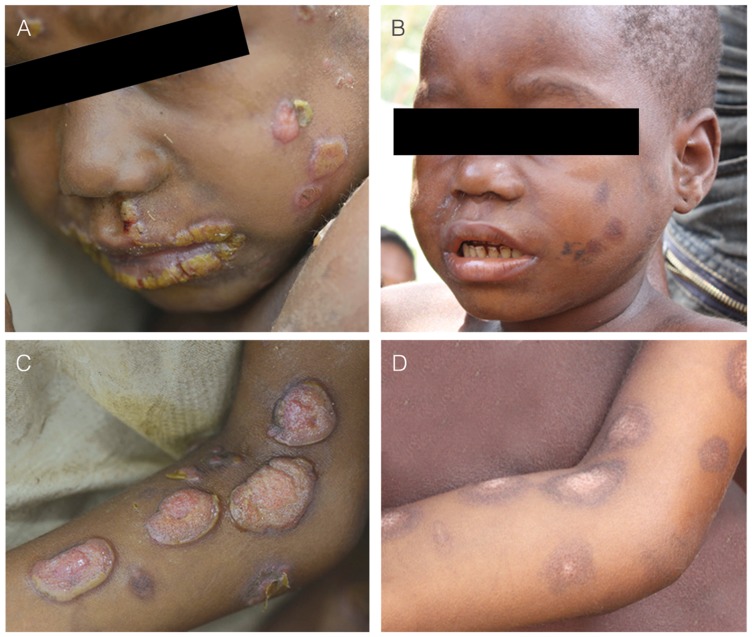
Results of treatment with a single dose of oral azithromycin in 2012. Panels A and C show a patient with yaws lesions (papilloma) on the face and arm before treatment. Panels B and D show the same patient three weeks after treatment with a single dose of azithromycin (courtesy of Mr. Lam Duc Hien and MSF-Epicentre, Paris, France).

In January 2012, the *Lancet* [41] published the results of the first study in Papua New Guinea, which showed that a single dose of azithromycin (Figure 6) was as effective in treating yaws as a single injection with benzathine penicillin. This finding signalled a major advance in the history of yaws control in the past 60 years and further contributed to reviving interest in a global eradication campaign. Azithromycin has been used extensively in mass treatment campaigns in the elimination of blinding trachoma, and its safety is well documented.

In March 2012, WHO organized a first consultation in Morges, Switzerland to develop a new yaws eradication strategy based on azithromycin [42]. This meeting resulted in the recommendation of two new treatment policies to replace those of the 1950s. The rationale of the new policies is to simplify the criteria for determining the prevalence of active yaws to guide treatment and ensure that incubating and latent infections are adequately dealt with. The two treatment policies are:

1. Total community treatment (TCT): treatment of an entire endemic community, irrespective of the number of active clinical cases.
2. Total targeted treatment (TTT): treatment of all active clinical cases and their contacts (household, school, and playmates).

The new policies recommend a first round of TCT with treatment coverage>90% (based on historic experience), followed by mop ups and active surveillance. Depending on the initial coverage, TTT rounds every six months may be adopted to actively detect and treat the remaining cases. Both theoretical and empirical studies are under way to validate the new strategy. Mathematical models will be useful to investigate the number of rounds of TCT that would need to be administered, and at what level of treatment coverage, in order to interrupt transmission.

In addition, to prove the feasibility of interrupting transmission using empirical data, the meeting recommended that pilot studies be conducted in one district each in six initial countries: Cameroon, Ghana, Indonesia, Papua New Guinea, the Solomon Islands, and Vanuatu. Later on, the Congo was added because of the intervention by Médecins Sans Frontières (MSF).

In March 2013, WHO organized a meeting of experts to prepare the guidelines for national programmes to implement the Morges Strategy and procedures for verification of transmission and eventual certification of countries [43], [44].

## Progress

As a first step in implementing the new eradication strategy, pilot treatment campaigns have been carried out in Congo (Bétou and Enyellé districts), Papua New Guinea (Lihir island), Vanuatu (Tafea Province), and Ghana (West Akyem district) in 2012 and 2013 to demonstrate the feasibility of eradicating the disease. About 90,000 people have been treated and coverage has exceeded 90%. One year follow-up results are expected. The community acceptability, especially children, of oral azithromycin (to replace painful injections) has been high (unpublished results). Like observed in the 1950s with penicillin, the rapid disappearance of the yaws lesions after a single-dose treatment with azithromycin reinforced community cooperation and encouraged those who missed the initial treatment to come forward to take their medication. The preliminary serological results indicate that the underlying prevalence of *T. pallidum* subsp. *Pertenue*; infection (active and latent) in some of these communities may be quite high 10%–30%. The proposed use of TCT approach is strongly supported by this evidence. A prevalence serological and clinical survey in combination with trachoma was also completed in the Solomon Islands in November 2013. Plans are under way to start large-scale treatment in 2014 in Cameroon, Indonesia, and the Solomon Islands.

In March 2014, WHO convened a third consultative meeting on yaws at its headquarters in Geneva, Switzerland. Detailed reports of the pilot implementation of the Morges strategy showed that treatment using TCT policy with azithromycin is feasible and can be carried out in different geographical areas. The results of the evaluation of the new dual POC syphilis test in Ghana, Papua New Guinea, the Solomon Islands, and Vanuatu confirmed that the test can accurately diagnose active and untreated yaws [45]. The new POC test results in the ability to screen and confirm the serological status of patients in the field and to reliably target yaws cases and contacts. Despite improved ability for diagnosis, programme managers still need to keep in mind diagnostic challenges of other causes of skin ulcers in the community, which may be confused with ulcerative yaws and which may not respond to azithromycin, giving the erroneous the impression that the treatment did not work. Recent reports show that ulcers caused by *Haemophilus ducreyi* co-exist in yaws-endemic areas and are a possible confounder of yaws diagnosis [46]. Results of studies on the baseline azithromycin resistance showed the absence of A2058G/A2059G point mutations on 23S rRNA in TPE strains, which implies no resistance, and azithromycin can be used. As recommended by the ITFDE in 2012, the meeting agreed to a proposal to conduct a clinical trial to clarify the different dosages of azithromycin for yaws and trachoma.

In the final phase of eradication, the use of molecular tools such as PCR may be necessary to confirm yaws in serologically-positive cases. Plans are at an advanced stage to build capacity in selected reference laboratories to support yaws eradication efforts.

## Investment Benchmarks for Yaws Eradication

In an accompanying paper on cost-effectiveness, we estimate that 21–74 million people will need treatment in the 12 endemic countries. The average dose per person treated is three 500 mg tablets. Including buffer stock and mop-up, no more than 92 million grams of azithromycin would be required during 2015–2020. This represents up to a quarter of the more than 375 million doses (up to 375 million grams) of Zithromax that has been donated by Pfizer towards the Global Elimination of Blinding Trachoma by 2020 (GET 2020) [47].

WHO has procured limited quantities of generic azithromycin (Medopharm, India) at US$0.17 (500 mg tablet) to kick-start the pilot projects. At this price, the cost of medicine would be about US$30 million. WHO has also purchased rapid dual nontreponemal and treponemal POC syphilis tests (from Chembio Diagnostic System Inc., New York, U.S.) at a negotiated price of US$2 per test to support the pilot implementation. At this price, the cost of diagnostic supplies adds about US$1 million. Surveillance is thought to add about US$20 million. The cost of surveillance in the 71 countries requiring verification of the absence of transmission of the disease is about US$30 million.

Based on the experience of the programmes in the Congo, Ghana, Lihir, and Tafea, as well as of mass drug administration campaigns for other NTDs, the cost of delivery is estimated at about US$300 million. The total cost is estimated at about US$360 million in the 12 known endemic countries. Excluding medicine, the cost is US$330 million. These are economic costs. Excluding existing Ministry of Health staff and assets, we estimate that the financial cost of yaws eradication (excluding medicine) could be as little as US$100 million in the 12 endemic countries.

By comparison, smallpox eradication is estimated to have cost US$300 million or about $23 million per year between 1967 and 1979 [48]. About US$125 million was spent on eradicating dracunculiasis to 2004; at least another US$191 million has been committed since then for the final push to 2015 [49], [50], [51]. Poliomyelitis eradication cost at least US$10,000 million between 1988 and 2012; another US$50,000 million are thought to be required to finish the job by 2018 [52].

The cost of the “end game” of any eradication effort is uncertain, with the emergence of complexities requiring some local adaptation of global strategies [53], [54]. Yaws elimination in India used rumor investigation, including cash rewards for the reporting of (subsequently confirmed) cases. In Tafea province, Vanuatu, communication-for-behavioral-impact (COMBI) was deemed necessary not only to improve acceptance rates, but also to improve general hygiene. Nonetheless, there are reasons to believe that yaws eradication can be achieved with a relatively modest investment in the period 2015–2020.

## Collaborations

The renewed eradication effort has seen the collaboration between WHO, ministries of health of the endemic countries, and the Centers for Disease Control and Prevention, Atlanta, U.S., University of Washington, Seattle, U.S., London School of Hygiene and Tropical Medicine, London, UK, Komfo Anokye Teaching Hospital, Kumasi, Ghana, Noguchi Memorial Institute for Medical Research, Accra, Ghana, Papua New Guinea Institute of Medical Research, and Barcelona Institute of Global Health, Barcelona, Spain.

## Conclusion

Since the 1950s, there have been seven eradication programs: hookworm, yellow fever, yaws, malaria, smallpox, dracunculiasis, and poliomyelitis [55]. Only smallpox eradication has been successful to date. Polio and dracunculiasis eradication programs are in their final stages but challenges remain [56].

Despite some concerns about eradication programs in general [57], [58], and the possibility that animal reservoirs for human treponemal pathogens may exist, the recent achievement in India has shown that eradication of yaws in modern times is technically and operationally feasible. Yearly clinical and serological surveys conducted between 2006 and 2013 have not identified any evidence of transmission in the country [59]. In today's environment, yaws eradication is technologically easier, and enthusiasm is growing. The probability of finally succeeding in this endeavor is greatly increased by the availability of a new, easier, and more effective treatment option and new diagnostic tools [60], [61], [62]. Single-dose treatment with azithromycin (oral) given in one or two rounds of large-scale treatment, depending on the initial coverage, may be sufficient to interrupt transmission. Post-treatment active surveillance will be enhanced by our ability to test any suspected case using the rapid dual POC treponemal and nontreponemal syphilis tests, which require only a finger prick drop of blood. Molecular testing using PCR can be used to definitively confirm yaws and to detect mutations conferring resistance to azithromycin from swab lesional specimens. Other favourable conditions for yaws eradication are that humans remain the most important reservoir for human transmission, relatively reduced geographical extent, and the rich historical experience with mass treatment campaigns to eradicate the disease. More recently, a lot of experience has accumulated with mass drug administration to control and eliminate helminthic infections including lymphatic filariasis, schistosomiasis, soil-transmitted helminthiases and onchocerciasis, and bacterial infections such as trachoma. Lessons from the past and current eradication programmes, including challenges, will guide the renewed yaws eradication effort, which is gaining momentum [63]. All considered, what is needed to achieve the WHO 2020 eradication target and end the human suffering caused by this easily curable disease is to find the goodwill, commitment, and necessary resources.

**Box 1. Recommendations of the International Task Force for Disease Eradication to WHO**

- Clarify the current geographical distribution of yaws.
- Prepare a provisional estimate of costs of the eradication.
- Stress the importance of health education and community mobilization in the yaws eradication strategy.
- Investigate the possible impact of mass drug administration of azithromycin for trachoma on yaws in co-endemic areas and research into the different dosages between yaws (30 mg/kg) and trachoma (20 mg/kg).
- Seek a donation of azithromycin.
- Monitor resistance to azithromycin.
- Clarify the epidemiological signficance of nonhuman primates in the transmission of yaws.
- Obtain a WHA resolution for yaws eradication.

**Box 2. Elimination of Yaws: India's Success Story**

- Realization that today's modern Indian society cannot have a disease like yaws that attacked a large proportion of the rural tribal population and incapacitating many.
- The fact that disease is cured by a single dose of treatment with a long-acting benzathine penicillin (now also with azithromycin) and can be eliminated.
- High-level political commitment, national ownership, vigorous and sustained implementation of the strategies, and local resources were pivotal to the success.
- Yaws elimination was included in the national health policy and a definite target date for completion was set.
- National Institute of Communicable Diseases led the elimination efforts.
- A National Task Force was established to monitor programme performance.
- The programme is evaluated from time to time by an independent body.

**Key Learning Points**

- India's recent yaws elimination success story provides motivation and demonstration that eradication of yaws is achievable.
- The disease is targeted for eradication by 2020 in World Health Assembly resolution WHA66.12 (2013).
- New technologies–single-dose oral azithromycin, new rapid point-of-care syphilis test, and molecular techniques–would facilitate the renewed eradication efforts.
- Unlike other mass treatments for NTDs, yaws requires limited rounds of large-scale treatment at intervals no longer than six months.

**Top Five Papers in the Field**

1. Ayove T, Houniei W, Wangnapi R, Bieb SV, Kazadi W, et al. (2014) Sensitivity and specificity of a rapid point-of-care test for active yaws: a comparative study. Lancet Glob Health 2: e415–421.
2. Mitjà O, Hays R, Ipai A, Penias M, Paru R, et al. (2012) Single-dose azithromycin versus benzathine benzylpenicillin for treatment of yaws in children in Papua New Guinea: an open-label, non-inferiority, randomised trial. Lancet 379: 342–347.
3. WHO (2012) Eradication of yaws – the Morges Strategy. Wkly Epidemiol Rec 87: 189–194.
4. WHO (2008) Elimination of yaws in India. Wkly Epidemiol Rec 83: 125–132.
5. Zahra A (1956) Yaws eradication campaign in Nsukka Division, Eastern Nigeria. Bull World Health Organ 15: 911–35.
